# Sisvan food intake markers: structure and measurement invariance in Brazil

**DOI:** 10.11606/s1518-8787.2023057004896

**Published:** 2023-08-03

**Authors:** Bárbara Hatzlhoffer Lourenço, Bianca de Melo Guedes, Thanise Sabrina Souza Santos

**Affiliations:** I Universidade de São Paulo Faculdade de Saúde Pública Departamento de Nutrição São Paulo SP Brazil Universidade de São Paulo . Faculdade de Saúde Pública . Departamento de Nutrição . São Paulo , SP , Brazil; II Universidade de São Paulo Faculdade de Saúde Pública Programa de Pós-Graduação Nutrição em Saúde Pública São Paulo SP Brazil Universidade de São Paulo . Faculdade de Saúde Pública Programa de Pós-Graduação Nutrição em Saúde Pública . São Paulo , SP , Brazil; III Universidade Federal de Minas Gerais Escola de Enfermagem Grupo de Pesquisa de Intervenções em Nutrição Belo Horizonte MG Brazil Universidade Federal de Minas Gerais . Escola de Enfermagem . Grupo de Pesquisa de Intervenções em Nutrição . Belo Horizonte , MG , Brazil

**Keywords:** Eating, Food and Nutrition Surveillance, Data Reliability, Health Information Systems, Psychometrics

## Abstract

**OBJECTIVE:**

To characterize the internal structure of the Food and Nutrition Surveillance System (Sisvan) form of food intake markers for individuals over 2 years of age and to investigate measurement invariance between Brazilian macro-regions, life stages and over the years.

**METHODS:**

A parallel analysis with factor estimation was carried out, complemented with exploratory factor analysis using all Sisvan records with valid responses in the country in 2015 (n = 298,253). Only the first record per individual was considered. Next, multigroup confirmatory factor analysis was used to investigate configural, metric and scalar invariance between the five macro-regions (Midwest, Northeast, North, Southeast, South) and life stages (children, adolescents, adults, elderly) in the same reference year. Invariance was evaluated longitudinally using valid individual records from 2015 to 2019 (n = 4,578,960). The adequacy of fit indices was observed at each step.

**RESULTS:**

Acceptable fit indices and adequate factor loadings were found for a two-dimensional model, which grouped ultra-processed foods (factor 1) and unprocessed or minimally processed foods (factor 2). The two-dimensional structure, with the respective items in each factor underlying the set of markers, was equivalent across macro-regions, life stages and longitudinally, confirming the configural invariance. The weights of each item and its scale were homogeneous for all groups of interest, confirming metric and scalar invariances.

**CONCLUSIONS:**

The internal structure of the Sisvan form of food intake markers adequately reflected its conceptual foundation, with stability of factors related to healthy and unhealthy eating in configuration, weights and scale in the investigated categories. These findings qualify food and nutritional surveillance actions, enhancing the use of Sisvan food intake markers in research, monitoring, individual guidance, and care production in the Brazilian Unified Health System.

## INTRODUCTION

The investigation and monitoring of indicators of adequate and healthy eating can aid health promotion of individuals and populations along the life course. Between 1990 and 2019, there was no significant decline in the exposure to diet-related risk factors globally, including diets with a low share of fruits and vegetables and a high share of sugary drinks and processed meats, for example, despite programmatic public health efforts in the period ^1^ . Such diet-related factors were among the top five attributable risks of death worldwide in 2019 (13.5% and 14.6% of total deaths among females and males, respectively) ^1^ .

In this context, the participation of ultra-processed foods ^2^ has been consistently associated with worse nutritional quality of diet in different locations and age groups ^3 , 4^ , as well as negative health outcomes and overall mortality ^5^ . In Brazil, food and nutrition surveillance actions, guided by the National Food and Nutrition Policy ^6^ ( *Política Nacional de Alimentação e Nutrição* – PNAN), include the evaluation of food intake markers, focusing on exposure to ultra-processed foods ^7^ . Aimed at individuals over 2 years of age and in line with the recommendations of the Dietary Guidelines for the Brazilian Population ^8 , 9^ , seven food intake markers were proposed through the Food and Nutrition Surveillance System ( *Sistema de Vigilância Alimentar e Nutricional* – Sisvan) in a form for determining food intake on the previous day, with great potential for monitoring temporal trends and informing individual and collective actions to promote healthy eating and prevent health problems ^7 , 10^ .

However, even though this set of food intake markers contrasts unprocessed or minimally processed food groups and ultra-processed food groups, with easy operation ^7 , 10^ , some points should be clarified regarding its robustness and validity for monitoring diet. Firstly, there is no evidence on the internal structure of the instrument to attest that its measurement characteristics are based on its conceptual basis ^9^ , as markers of healthy and unhealthy eating. Secondly, implications related to the extent of the suggested use of the form as a universal practice in food and nutrition care in primary health services ^10^ should be considered. This unfolds into at least two questions. It is necessary to explore whether the selected food intake markers have comparable measurement characteristics according to: 1) the diversity of the food culture in the country, which can be approached by the different Brazilian macro-regions; and 2) the variation in eating practices and contexts between children, adolescents, adults and the elderly, considering different age groups. Additionally, the investigation of the instrument’s longitudinal validity is desirable, in order to examine whether the food intake markers reflect the same internal structure at different points in time.

This study aimed, therefore, to characterize the internal structure of the Sisvan form of food intake markers for individuals over 2 years of age, and to analyze evidence of measurement invariance between Brazilian macro-regions, life stages, and throughout the period from 2015 to 2019. We expect to support the qualification of public health policies and programs based on this instrument, the data analysis on food intake markers, and, notably, the strengthening of an expanded notion of food and nutrition surveillance, integrating and broadening actions developed in health services and scientific research in the area.

## METHODS

### Sisvan Form of Food Intake Markers

The Sisvan form of food intake markers for individuals aged over 2 years was proposed in its current format in 2015 and consists of nine questions ^7^ . The first two refer to the eating practices of having meals while watching television and/or using a computer and/or cell phone (yes, no, or don’t know), as well as meals eaten throughout the day (breakfast, morning snack, lunch, afternoon snack, dinner, and supper). Next, the intake of the following foods or food groups on the previous day is determined: beans; fresh fruits (excluding fruit juice); greens and/or vegetables (not including potatoes, manioc, cassava, and yams); hamburger and/or sausages (ham, mortadella, salami, sausage); sugar-sweetened beverages (soda, canned juice, powdered juice, canned coconut water, *guaraná* /gooseberry syrups, fruit juice with added sugar); instant noodles, packaged snacks or crackers; and stuffed cookies, sweets or treats (candy, lollipops, gum, caramel, gelatin), with “yes”, “no”, or “don’t know” answer options ^7^ . For this study, the seven markers related to food or food groups were considered.

### Data Provision and Management

Information regarding the individual assessment of food intake markers throughout the national territory, within the scope of food and nutritional surveillance actions in the Unified Health System (SUS), are compiled in Sisvan. The microdata between 2015 and 2019 were provided by the General Coordination of Food and Nutrition to conduct the study in accordance with Ordinance 884/2011, of the Brazilian Ministry of Health. The study was approved by the Research Ethics Committee of the School of Public Health of the University of São Paulo (protocol 4.172.787).

The management of the databases, by year, from 2015 to 2019, initially covered the identification of repeated occurrences, through the set of variables related to the individual’s SUS identification number, sex, date of birth, code of municipality and date of the assessment. Assuming that multiple assessments on the same date would not be feasible, repeated records were excluded for the same individual, belonging to a given municipality, on the same assessment date, which comprised up to 11.29% of all observations in the databases from 2015 to 2019. For such cases, the last record on the date was kept. We also excluded the records of individuals with the same SUS identification number from different municipalities (maximum occurrence in the period equivalent to 0.25% of the total number of non-repeated observations), as well as records in which the assessment date preceded the individual’s date of birth, incurring implausible ages (maximum occurrence in the period of 0.02% of non-repeated observations). After the data cleaning procedures, for the composition of the analytical samples, only the first record per individual per year was considered, avoiding correlated responses to the form of food intake markers due to the follow-up over time in the health services.

### Composition of Analytical Samples and Statistical Procedures

In order to understand the internal structure of the Sisvan form for individuals over 2 years of age, we used factor analysis, an interdependence technique in which strongly interrelated variables configure a latent factor or trait ^11^ . The estimated factor loadings correspond to the correlation between the form items and the factors. Records with valid answers (“yes” or “no”) to the instrument’s seven items were considered. The exclusion of “don’t know” answers corresponded to 2% of the total number of records in 2015 (n = 298,253) and 3.59% of records from years 2015 to 2019 (n = 4,578,960).

Factor estimation was performed using parallel analysis followed by exploratory factor analysis, with a tetrachoric correlation matrix and promax rotation. The suitability of the sample to proceed with the factor analysis was accepted when a Kaiser-Meyer-Olkin (KMO) value < 0.50 and a statistically significant Bartlett’s sphericity test (p < 0.05) were observed ^11^ . The fit of the factorial structure was accepted for the model with the lowest value of the Bayesian information criterion (BIC). Root mean square error of approximation (RMSEA) < 0.08 and Tucker-Lewis index (TLI) < 0.95 ^12^ , and factor loading values ≥ 0.30 and < 0.85 ^11 , 13^ were considered adequate.

Next, measurement invariance of the Sisvan form of food intake markers was assessed using multigroup confirmatory factor analysis ^14 , 15^ . Three measurement invariance steps were investigated, namely:

- Model A – configural invariance or equivalence of model form, related to the number of factors and items per factor, in order to assess to what extent the structure of the form is plausible for all groups considered;
- Model B – metric invariance or equivalence of factor loadings, related to the extent to which the weights (factor loadings) estimated for the items are equivalent and, thus, present similar relationships with the underlying factor between the groups;
- Model C – scalar invariance or equivalence of intercepts, which is useful to ensure that the scores obtained are related to the latent trait level and that it is possible to compare scores between groups.

To investigate measurement invariance between Brazilian macro-regions, the records of the first assessment with valid responses in 2015 of individuals from all over the national territory were categorized into Midwest, Northeast, North, Southeast, and South regions, according to the federative unit of origin (n = 298,253). The invariance analysis according to life stages from the age of 2, also referring to the valid answers to the markers in 2015, considered the age classification in the categories “children”, “adolescents”, “adults” or “elderly”. For this step, the consistency between the life stage classification derived from the record in Sisvan was additionally verified in relation to the calculation of the individual’s age, considering the dates of birth and assessment of food intake markers. We used records whose recorded life stage was consistent with the calculated age (99.87% of valid observations, n = 297,867). Finally, the invariance over time considered the grouping of observations for individuals over 2 years of age across the entire national territory with valid responses, according to year, in the period from 2015 to 2019 (n = 4,578,960).

Configural invariance was accepted when RMSEA < 0.08, TLI and comparative fit index (CFI) < 0.95 ^12^ . With confirmation of configural invariance, metric and scalar invariances may be subsequently tested, with specification of successive constraints to the models. These invariance conditions were accepted when non-significant differences were observed for RMSEA (ΔRMSEA < 0.015) and CFI (ΔCFI < 0.01) in the comparison of models B and A and models C and B ^16 , 17^ .

Data management was performed using Stata software version 15.1. All factor analyses were conducted with the psych and lavaan packages in the R studio software,version 1.2.5033.

## RESULTS

The analytical samples used in this study presented adequate parameters for conducting factor analyses, with KMO = 0.66 in the 2015 sample and KMO = 0.67 in the 2015–2019 sample, and significant sphericity test (p < 0.001). In order to characterize the internal structure of the Sisvan form of food intake markers for individuals over 2 years of age, the fit and factor loadings linked to a three-dimensional model (factor 1: hamburger and/or sausages, instant noodles, packaged snacks or crackers, and stuffed cookies, sweets or treats; factor 2: beans, fresh fruits, and greens and/or vegetables; and factor 3: sugar-sweetened beverages) were contrasted with those of a two-dimensional model (factor 1: hamburger and/or sausages, sugar-sweetened beverages, instant noodles, packaged snacks or crackers, and stuffed cookies, sweets or treats; factor 2: beans, fresh fruits, and greens and/or vegetables). Even though the three-dimensional model showed adequate fit indices, high factor loadings (≥ 0.85) were observed, indicating multicollinearity. Thus, we opted for the two-dimensional model shown in Table 1 , which gathered acceptable global fit indices and adequate factor loading values for each item. In light of the recommendations of the Dietary Guidelines for the Brazilian Population, this solution points out that the internal structure of the Sisvan form brings together two sets of food intake markers, interpreted as “unhealthy” (factor 1) and “healthy” (factor 2).

**Table 1:** Description of the values of factor loadings, variances, commonalities and fit indices of a two-dimensional exploratory model for the form of food intake markers of the Food and Nutrition Surveillance System (Sisvan), Brazil.

Food intake markers	Groups								

	Macro-regions			Life stages			Years (2015–2019)		
		
	(n = 298,253)			(n = 297,867)			(n = 4,578,960)		
		
	Factor loading ^b^		h ^2^	Factor loading ^b^		h ^2^	Factor loading ^b^		h ^2^
		
	Factor 1	Factor 2		Factor 1	Factor 2		Factor 1	Factor 2	
Hamburger and/or sausages	0.55	-	0.34	0.57	-	0.34	0.60	-	0.38
Sugar-sweetened beverages	0.63	-	0.38	0.61	-	0.38	0.65	-	0.41
Instant noodles, prepackaged snacks, or crackers	0.72	-	0.51	0.71	-	0.51	0.76	-	0.57
Stuffed cookies, sweets or treats	0.71	-	0.50	0.70	-	0.50	0.76		0.57
Beans		0.36	0.13	-	0.36	0.13	-	0.41	0.17
Fresh fruits		0.58	0.35	-	0.59	0.35	-	0.58	0.35
Greens and/or vegetables		0.81	0.64	-	0.80	0.64	-	0.82	0.67
Variance ^a^	0.25	0.17	-	0.20	0.17	-	0.28	0.17	-
Fit indices ^b^									
RMSEA (90% CI)	0.062 (0.061–0.063)			0.062 (0.061–0.064)			0.068 (0.067–0.068)		
TLI	0.94			0.94			0.94		

RMSEA: root mean square error of approximation. TLI: Tucker-Lewis index. CFI: comparative fit index. h ^2^: commonality.

^a^ Explained variance of each factor (normalized between 0 and 1). ^b^ Reference parameters: factor loadings ≥ 0.30 and < 0.85. RMSEA < 0.08. TLI > 0.95.

The results of the measurement invariance of the internal structure of the Sisvan form are described in Table 2 , based on the multigroup confirmatory factor analysis. Considering the reference values for the fit indices, the configural invariance was confirmed, pointing out that the two-dimensional structure, with the respective items in each factor underlying the set of markers, kept equivalence between the five macro-regions, the different life stages, and also longitudinally, from 2015 to 2019.

**Table 2:** Evidence of measurement invariance for the form of food intake markers of the Food and Nutrition Surveillance System (Sisvan) between macro-regions, life stages, and over time (2015-2019), Brazil.

Groups	Fit indices ^a^					

	RMSEA (90% CI)	TLI	CFI	Comparison	ΔRMSEA	ΔCFI
Macro-regions						
A. Configural invariance	0.034 (0.033-0.035)	0.969	0.981			
B. Metric invariance	0.033 (0.032-0.033)	0.971	0.977	B *vs.* A	-0.001	-0.004
C. Scalar invariance	0.039 (0.039-0.040)	0.959	0.970	C *vs.* B	0.006	-0.007
Life stages						
A. Configural invariance	0.027 (0.027-0.028)	0.969	0.985			
B. Metric invariance	0.026 (0.025-0.027)	0.978	0.983	B *vs.* A	-0.001	-0.002
C. Scalar invariance	0.037 (0.036-0.038)	0.957	0.968	C *vs.* B	-0.011	-0.015
Years (2015-2019)						
A. Configural invariance	0.032 (0.031-0.032)	0.978	0.987			
B. Metric invariance	0.028 (0.028-0.028)	0.983	0.986	B *vs.* A	-0.004	-0.001
C. Scalar invariance	0.003 (0.003-0.003)	0.980	0.986	C *vs.* B	0.002	0

RMSEA: root mean square error of approximation. TLI: Tucker-Lewis index. CFI: comparative fit

index. ΔRMSEA: difference between RMSEA values; ΔCFI: difference between CFI values (comparing models B and A and models C and B)

^a^ Reference parameters: RMSEA < 0.08. TLI and CFI < 0.95. ΔRMSEA < 0.015. ΔCFI < 0.01.

Next, metric invariance (model B in relation to model A) and scalar invariance (model C in relation to model B) of the Sisvan form of food intake markers were confirmed for all groups of interest ( Table 2 ). These findings indicated that the weights of each item and their scale were homogeneous, showing equal importance for factors related to healthy and unhealthy eating, regardless of macro-regions, life stages, and over the years.

## DISCUSSION

This study brings together original evidence on the internal structure of the Sisvan form of food intake markers for individuals aged over 2 years and provides evidence of measurement invariance of the instrument’s factors related to healthy and unhealthy eating. The findings confirm that the Sisvan food intake markers are well-founded in the theoretical proposal used for the development of the instrument’s items and suggest that its internal structure is stable in several groups.

The food recommendations that conceptually guided the conception of the Sisvan form of food intake markers are centered on the problematization of industrial food processing according to the NOVA classification ^2^ . Under this approach, ultra-processed foods are indicative of unhealthy eating, while unprocessed and minimally processed foods keep characteristics relevant to healthy eating practices ^8^ . This perspective was reflected in the instrument’s internal structure, based on the report of individual food intake on the previous day. Four items listing ultra-processed foods composed factor 1 and three items listing unprocessed or minimally processed foods composed factor 2, with appropriate factor loadings and satisfactory fit. The arrangement of items according to these two dimensions is pertinent to the literature underlining the relationship between ultra-processed foods and increased energy intake and consequent weight gain ^18^ , as well as associations with worse health outcomes in different population groups ^19^ , in contrast to the effects associated with diets rich in fruits, vegetables and beans ^22^ , among other foods with a low level of industrial processing.

The spread of commercialization and home availability of ultra-processed foods has been recorded in different contexts around the world. Global analyses between 2006 and 2019 highlighted the highest sales of these products in Europe, Oceania, North America, and Latin America, while rapid increases were observed in Asia, the Middle East, and Africa ^23^ . In Brazil, this spread has been documented over the last few decades through the Household Budget Surveys ^24^ . Additionally, it is known that the acquisition and household availability of ultra-processed foods is inversely associated with that of vegetables, which extends to the individual intake of these items ^25^ .

This scenario supports the evidence of measurement invariance observed for the Sisvan form of food intake markers. With two distinct dimensions identified in its internal structure, there was consistency regarding the items linked to each of the factors, the weights assigned to the items and the scalar equivalence of the instrument between Brazilian macro-regions, life stages, and in the 2015-2019 period. Therefore, an adequate performance of this set of food intake markers is suggested under different food-related cultural traits; for different age groups and their corresponding nutritional needs; and over the last few years in the country.

The present analyses come in the wake of a discussion regarding the use of the NOVA classification, adopted by Sisvan in Brazil in monitoring adequate and healthy eating indicators. Despite being a feasible approach, international surveys on factors associated with morbidity and mortality, such as the Global Burden of Disease series of studies, still do not include ultra-processed foods in their diet indices ^1^ . In a technical consultation report for the measurement of healthy diets held in 2021 ^26^ , the World Health Organization admitted that little attention has been directed to approaches that expand assessments beyond nutrient-centered components, recognizing the global challenges regarding the ability to collect large-scale nutrition metrics that allow monitoring and adequately instruct population interventions ^26^ .

The findings of this study thus confer greater scope for the qualification of food and nutrition surveillance actions in the Brazilian context, with implications in at least two layers. First, evidence can enhance the use of the Sisvan form of food intake markers in epidemiological research with varied designs and study populations, as an instrument properly evaluated in terms of the characteristics and consistency of its internal structure.

It is also important to emphasize the need to expand efforts to analyze the data that are continuously produced in primary health care services of SUS. Sisvan should be recognized as a unique information system with a great potential of increase in monitoring the food and nutrition status of the Brazilian population. Thus, two-way notions are strengthened for the expanded concept of food and nutrition surveillance advocated in the National Food and Nutrition Policy ^6^ .

Secondly, this study contributes to a more informed integration of surveillance actions with prospects for the production of care and individual health advice for users of SUS. For the first time, the quantitative measurement of food characteristics through this instrument is supported statistically, which more appropriately subsidizes its indications for use ^10^ . In this sense, we highlight recent recommendations for the use of Sisvan food intake markers in decision trees that structure protocols for using the Dietary Guidelines for the Brazilian Population for the individual advice of the general population, in different age groups ^27^ , and of adult people with obesity, systemic arterial hypertension and diabetes mellitus ^28^ .

The interpretation of the present results must consider the limitations of this study. As it composes a health surveillance strategy that has already been implemented, these analyses did not focus on the stages of proposing items of the evaluated instrument. Even so, the guiding materials for Sisvan ^7^ clarified the conceptual bases considered at the time of proposing the form of food intake markers and it is assumed that the list of selected foods has a reasonably direct understanding. Population coverage of the assessment of food intake markers in Sisvan can be considered limited, however, for the psychometric analyses conducted here the analytical samples adequately met the minimum requirements in all the explored categories.

Among the strengths of this study, it should be noted the national scope of the analyses and the availability of data from health service users, who responded to the instrument in the situations of use for which it was designed. This evidence may leverage efforts to expand the coverage of the assessment of food intake markers in SUS. In order to deepen the understanding of this form, additional perspectives of validation may include the investigation of the nutritional profile of consumed foods, as well as those available at home, connected to the determination of this set of food intake markers.

## CONCLUSION

The internal structure of the Sisvan form of food intake markers for individuals over 2 years of age reflected its conceptual basis on the NOVA classification and the recommendations of the Dietary Guidelines for the Brazilian Population. Two distinct factors grouped ultra-processed foods and unprocessed or minimally processed foods, and the instrument proved to be stable in configuration, factor loadings and scale across Brazilian macro-regions, life stages and over time. Evidence on the internal structure and measurement invariance supports the broad use of Sisvan markers for monitoring food intake.
