# Supplementary material for: Sisvan food intake markers: structure and measurement invariance in Brazil
Source: Rev Saude Publica. 2023 Aug 3;57:52. doi: 10.11606/s1518-8787.2023057004896 (PMC10421608; doi:10.11606/s1518-8787.2023057004896)
Supplement: Translation

# Translation: Marcadores do consumo alimentar do Sisvan: estrutura e invariância de mensuração no Brasil

**Authors:** Bárbara Hatzlhoffer Lourenço, Bianca de Melo Guedes, Thanise Sabrina Souza Santos

**Affiliations:** I Brazil Universidade de São Paulo. Faculdade de Saúde Pública. Departamento de Nutrição. São Paulo, SP, Brazil; II Brazil Universidade de São Paulo. Faculdade de Saúde Pública Programa de Pós-Graduação Nutrição em Saúde Pública. São Paulo, SP, Brazil; III Brazil Universidade Federal de Minas Gerais. Escola de Enfermagem. Grupo de Pesquisa de Intervenções em Nutrição. Belo Horizonte, MG, Brazil

**Keywords:** Ingestão de Alimentos, Vigilância Alimentar e Nutricional, Confiabilidade dos Dados, Sistemas de Informação em Saúde, Psicometria

## Abstract

**OBJETIVO:**

Caracterizar a estrutura interna do formulário de marcadores do consumo alimentar do Sistema de Vigilância Alimentar e Nutricional (Sisvan) para indivíduos maiores de 2 anos de idade e analisar evidências de invariância de mensuração entre macrorregiões brasileiras, fases do curso da vida e ao longo do tempo.

**MÉTODOS:**

Realizou-se análise paralela com estimação de fatores complementada com análise fatorial exploratória a partir de todos os registros de primeiro acompanhamento do Sisvan com respostas válidas no país em 2015 (n = 298.253). A seguir, empregou-se a análise fatorial confirmatória multigrupo para investigação de evidências de invariância configural, métrica e escalar entre as cinco macrorregiões (Centro-Oeste, Nordeste, Norte, Sudeste e Sul) e fases do curso da vida (crianças, adolescentes, adultos e idosos), no mesmo ano de referência. Avaliou-se a invariância longitudinalmente a partir de registros individuais válidos de 2015 a 2019 (n = 4.578.960). A adequação de índices de ajuste foi observada a cada etapa.

**RESULTADOS:**

Verificaram-se índices de ajuste aceitáveis e valores de carga fatorial adequados para um modelo bidimensional, que agrupou alimentos ultraprocessados (fator 1) e alimentos *in natura* ou minimamente processados (fator 2). A estrutura bidimensional, com os respectivos itens em cada fator subjacente ao conjunto de marcadores, foi equivalente entre macrorregiões, fases do curso da vida e longitudinalmente, confirmando a invariância configural. Os pesos de cada item e sua escala foram homogêneos para todos os grupos de interesse, confirmando as invariâncias métrica e escalar.

**CONCLUSÕES:**

A estrutura interna do formulário de marcadores do consumo alimentar refletiu adequadamente seu embasamento conceitual, com estabilidade dos fatores relacionados à alimentação saudável e não saudável em configuração, cargas e escala nos recortes investigados. Esses achados qualificam ações de vigilância alimentar e nutricional, potencializando o uso dos marcadores do consumo alimentar do Sisvan em pesquisas, monitoramento, orientação individual e produção de cuidado no Sistema Único de Saúde.

## INTRODUÇÃO

A investigação e o monitoramento de indicadores de alimentação adequada e saudável pode possibilitar a promoção da saúde de indivíduos e populações ao longo do curso da vida. Entre 1990 e 2019, a exposição a fatores de risco relacionados à alimentação, incluindo dietas com baixa participação de frutas e hortaliças e alta participação de bebidas açucaradas e carnes processadas, por exemplo, não recuou de maneira relevante em nível global, apesar dos esforços programáticos em saúde pública no período ^1^ . Tais fatores relacionados à alimentação estiveram entre os cinco principais riscos para mortes atribuíveis em todo o mundo em 2019 (13,5% e 14,6% do total de mortes entre indivíduos do sexo feminino e masculino, respectivamente) ^1^ .

Nesse contexto, a participação de alimentos ultraprocessados ^2^ tem sido consistentemente associada à pior qualidade nutricional da alimentação em diferentes localidades e grupos etários ^3 , 4^ , bem como a desfechos de saúde negativos e mortalidade geral ^5^ . No Brasil, as ações de vigilância alimentar e nutricional, pautadas pela Política Nacional de Alimentação e Nutrição ^6^ , abrangem um componente de avaliação de marcadores do consumo alimentar, com foco na exposição a alimentos ultraprocessados ^7^ . Dirigidos a indivíduos a partir de 2 anos de idade e alinhados às recomendações do Guia Alimentar para a População Brasileira ^8 , 9^ , sete marcadores foram propostos por meio do Sistema de Vigilância Alimentar e Nutricional (Sisvan) em um formulário para apuração do consumo alimentar referente ao dia anterior, com grande potencial para monitorar tendências temporais e informar ações em âmbito individual e coletivo para a promoção da alimentação saudável e a prevenção de agravos à saúde ^7 , 10^ .

No entanto, ainda que esse conjunto de marcadores contraste grupos de alimentos *in natura* ou minimamente processados e grupos de alimentos ultraprocessados, com fácil operacionalização ^7 , 10^ , alguns pontos devem ser elucidados quanto à sua robustez e à sua validade para o monitoramento da alimentação. Primeiramente, não existem evidências sobre a estrutura interna do instrumento que atestem características de mensuração, a partir de seu embasamento conceitual ^9^ , como marcadores de alimentação saudável e não saudável. Em segundo lugar, devem ser ponderadas implicações relacionadas à amplitude do emprego sugerido do formulário como prática universal no cuidado em alimentação e nutrição na atenção primária à saúde ^10^ , o que se desdobra ao menos em dois questionamentos. Faz-se necessário explorar se os marcadores selecionados têm características comparáveis de mensuração segundo: 1) a diversidade da cultura alimentar no país, que pode ser aproximada de forma sumária pelas distintas macrorregiões brasileiras; e 2) a variação de práticas e contextos alimentares entre crianças, adolescentes, adultos e idosos, considerando diferentes grupos etários. Adicionalmente, a investigação da validade longitudinal do instrumento é desejável, de maneira a examinar se os marcadores refletem a mesma estrutura interna em diferentes pontos no tempo.

O presente estudo objetivou, portanto, caracterizar a estrutura interna do formulário de marcadores do consumo alimentar do Sisvan para indivíduos a partir de 2 anos de idade, e analisar evidências de invariância de mensuração entre macrorregiões brasileiras, fases do curso da vida e ao longo do período de 2015 a 2019. Espera-se apoiar a qualificação de políticas e programas de interesse de saúde pública baseados nesse instrumento, a análise de dados referentes aos marcadores do consumo alimentar, e, sobretudo, o fortalecimento de uma noção ampliada do exercício de vigilância alimentar e nutricional, integrando e expandindo ações desenvolvidas em serviços de saúde a iniciativas de pesquisa científica na área.

## MÉTODOS

### Formulário de Marcadores do Consumo Alimentar do Sisvan

O formulário de marcadores do consumo alimentar do Sisvan para indivíduos a partir de 2 anos de idade foi proposto em seu formato atual em 2015 e é composto por nove questões ^7^ . As duas primeiras referem-se ao hábito de realizar refeições assistindo à televisão e/ou usando computador e/ou celular (sim, não e não sabe), bem como às refeições realizadas ao longo do dia (café da manhã, lanche da manhã, almoço, lanche da tarde, jantar e ceia). Na sequência, apura-se o consumo dos seguintes alimentos ou grupos de alimentos no dia anterior: feijão; frutas frescas (sem considerar suco de frutas); verduras e/ou legumes (sem considerar batata, mandioca, aipim, macaxeira, cará e inhame); hambúrguer e/ou embutidos (presunto, mortadela, salame, linguiça, salsicha); bebidas adoçadas (refrigerante, suco de caixinha, suco em pó, água de coco de caixinha, xaropes de guaraná/groselha, suco de fruta com adição de açúcar); macarrão instantâneo, salgadinhos de pacote ou biscoitos salgados; e biscoito recheado, doces ou guloseimas (balas, pirulitos, chiclete, caramelo, gelatina), com opções de resposta “sim”, “não” e “não sabe” ^7^ . Para este estudo, consideraram-se os sete marcadores relacionados a alimentos ou grupos de alimentos.

### Cessão e Gerenciamento de Dados

Informações relativas à avaliação individual de marcadores do consumo alimentar em todo o território nacional, no escopo de ações de vigilância alimentar e nutricional no Sistema Único de Saúde (SUS), são compiladas no Sisvan. Os microdados do Sisvan entre os anos de 2015 e 2019 foram cedidos pela Coordenação-Geral de Alimentação e Nutrição para condução do estudo de acordo com a Portaria nº 884/2011, do Ministério da Saúde. O estudo foi aprovado pelo Comitê de Ética em Pesquisa da Faculdade de Saúde Pública da Universidade de São Paulo (parecer n° 4.172.787).

O gerenciamento dos bancos de dados cedidos, por ano, no período de 2015 a 2019, abrangeu inicialmente a identificação de ocorrências repetidas, por meio do conjunto de variáveis relativas a número de identificação do indivíduo no SUS, sexo, data de nascimento, código do município e data de acompanhamento. Partindo do pressuposto de que não seriam factíveis avaliações múltiplas em uma mesma data de acompanhamento, foram excluídos registros repetidos para um mesmo indivíduo, pertencente a um dado município, em uma mesma data de acompanhamento, os quais compuseram até 11,29% de todas as observações nos bancos de dados de 2015 a 2019. Para tais casos, o último registro na data foi mantido. Optou-se, ainda, pela exclusão de registros de avaliações de indivíduos com mesmo número de identificação no SUS em municípios distintos (ocorrência máxima no período equivalente a 0,25% do total de observações não repetidas), bem como de registros em que a data de acompanhamento antecedia a data de nascimento do indivíduo, incorrendo em idades implausíveis (ocorrência máxima no período de 0,02% das observações não repetidas). Após os procedimentos de limpeza de dados, para composição das amostras analíticas foi considerado apenas o primeiro registro por indivíduo, por ano, evitando-se respostas correlacionadas ao formulário de marcadores do consumo alimentar devido ao acompanhamento ao longo do tempo nos serviços de saúde.

### Composição de Amostras Analíticas e Procedimentos Estatísticos

Para compreensão da estrutura interna do formulário do Sisvan para indivíduos a partir de 2 anos de idade, foi empregada a análise fatorial, uma técnica de interdependência em que variáveis fortemente inter-relacionadas se configuram em um fator ou traço latente ^11^ . As cargas fatoriais estimadas correspondem à correlação entre os itens do formulário e os fatores. Foram considerados registros com respostas válidas (“sim” ou “não”) aos sete itens do instrumento. A exclusão de respostas “não sabe” correspondeu a 2% do total de registros em 2015 (n = 298.253) e a 3,59% do total no agrupamento dos anos de 2015 a 2019 (n = 4.578.960).

Realizou-se a estimação de fatores por meio de análise paralela seguida por análise fatorial exploratória, com matriz de correlação tetracórica e rotação promax. A adequação da amostra para proceder com a análise fatorial foi aceita quando observados valor de Kaiser-Meyer-Olkin (KMO) < 0,50 e teste de esfericidade de Bartlett estatisticamente significante (p < 0,05) ^11^ . O ajuste da estrutura fatorial foi aceito para o modelo com menor valor do critério *Bayesian information criterion* (BIC), sendo considerados adequados os valores de *root mean square error of approximation* (RMSEA) < 0,08 e de *Tucker-Lewis index* (TLI) < 0,95 ^12^ . Ademais, foram considerados adequados os valores de cargas fatoriais ≥ 0,30 e < 0,85 ^11 , 13^ .

Na sequência, para o estudo da invariância da estrutura do formulário de marcadores do consumo alimentar do Sisvan, foi empregada a análise fatorial confirmatória multigrupo ^14 , 15^ . Três evidências de invariância de mensuração foram investigadas, a saber:

- Modelo A – invariância configural ou de igualdade de forma, relacionada ao número de fatores e itens por fator, de maneira a avaliar em que medida a estrutura do formulário é plausível para todos os grupos considerados;
- Modelo B – invariância métrica ou de igualdade de cargas fatoriais, relacionada à medida em que os pesos (cargas fatoriais) estimados para os itens são equivalentes e, assim, apresentam relações semelhantes com o fator subjacente entre os grupos;
- Modelo C – invariância escalar ou de igualdade de interceptos, útil para garantir que os escores obtidos estejam relacionados com o nível de traço latente e que seja possível a comparação de escores entre os grupos.

Para investigação da invariância entre macrorregiões brasileiras, os registros do primeiro acompanhamento de indivíduos de todo o território nacional com respostas válidas em 2015 foram categorizados em regiões Centro-Oeste, Nordeste, Norte, Sudeste e Sul, de acordo com a unidade federativa de procedência (n = 298.253). As análises de invariância de acordo com as fases do curso da vida, a partir dos 2 anos de idade, consideraram a classificação etária nas categorias “crianças”, “adolescentes”, “adultos” e “idosos”, também referentes às respostas válidas aos marcadores em 2015. Para essa etapa, a consistência entre a classificação de fase de vida advinda do registro no Sisvan foi adicionalmente verificada em relação ao cálculo da idade do indivíduo, considerando as datas de nascimento e acompanhamento registradas. Optou-se pela utilização de registros cuja fase de vida registrada era concordante com a idade calculada (99,87% das observações válidas, n = 297.867). Finalmente, a invariância ao longo do tempo considerou o agrupamento de observações para indivíduos a partir de 2 anos de todo o território nacional com respostas válidas, segundo ano, no período de 2015 a 2019 (n = 4.578.960).

A invariância configural foi aceita quando RMSEA < 0,08, TLI e *comparative fit index* (CFI) < 0,95 ^12^ . Com a confirmação das condições de invariância configural, pode-se testar subsequentemente as invariâncias métrica e escalar, com a especificação de restrições sucessivas aos modelos. Tais condições de invariância foram aceitas quando observadas diferenças não significativas para RMSEA (ΔRMSEA < 0,015) e CFI (ΔCFI < 0,01) na comparação dos modelos B e A e dos modelos C e B ^16 , 17^ .

O gerenciamento dos dados foi realizado no software Stata versão 15.1. Todas as análises fatoriais foram conduzidas com os pacotes *psych* e *lavaan* no software R studio, versão 1.2.5033.

## RESULTADOS

As amostras analíticas utilizadas neste estudo apresentaram parâmetros adequados para realização das análises fatoriais, com KMO = 0,66 na amostra correspondente a 2015 e KMO = 0,67 no agrupamento dos anos de 2015 a 2019, e teste de esfericidade significante (p < 0,001). Para caracterização da estrutura interna do formulário de marcadores do consumo alimentar do Sisvan para indivíduos a partir de 2 anos de idade, o ajuste e as cargas fatoriais atreladas a um modelo de três dimensões (fator 1: hambúrguer e/ou embutidos, macarrão instantâneo, salgadinhos de pacote ou biscoitos salgados, e biscoito recheado, doces ou guloseimas; fator 2: feijão, frutas frescas, e verduras e/ou legumes; e fator 3: bebidas adoçadas) foram contrapostos com aqueles de um modelo de apenas duas dimensões (fator 1: hambúrguer e/ou embutidos, bebidas adoçadas, macarrão instantâneo, salgadinhos de pacote ou biscoitos salgados, e biscoito recheado, doces ou guloseimas; fator 2: feijão, frutas frescas, e verduras e/ou legumes). Ainda que o modelo com três dimensões tenha apresentado índices de ajuste adequados, foram observadas cargas fatoriais elevadas (≥ 0,85), indicativas de multicolinearidade. Dessa forma, optou-se pelo modelo bidimensional apresentado na Tabela 1 , que reuniu índices globais de ajuste aceitáveis e valores de carga fatorial para cada item adequados. À luz das recomendações do Guia Alimentar para a População Brasileira, esta solução aponta que a estrutura interna do formulário do Sisvan reúne dois conjuntos de marcadores do consumo alimentar, interpretados como “não saudável” (fator 1) e “saudável” (fator 2).

**Tabela 1:** Descrição dos valores de cargas fatoriais, variâncias, comunalidades e índices de ajustes de modelo exploratório bidimensional para o conjunto de marcadores do consumo alimentar do Sistema de Vigilância Alimentar e Nutricional (Sisvan), Brasil.

Marcadores do consumo alimentar	Grupos								

	Macrorregiões			Fases do curso da vida			Anos (2015–2019)		
		
	(n = 298.253)			(n = 297.867)			(n = 4.578.960)		
		
	Carga fatorial ^b^		h ^2^	Carga fatorial ^b^		h ^2^	Carga fatorial ^b^		h ^2^
		
	Fator 1	Fator 2		Fator 1	Fator 2		Fator 1	Fator 2	
Hambúrguer e/ou embutidos	0,55	-	0,34	0,57	-	0,34	0,60	-	0,38
Bebidas adoçadas	0,63	-	0,38	0,61	-	0,38	0,65	-	0,41
Macarrão instantâneo, salgadinhos de pacote ou biscoitos salgados	0,72	-	0,51	0,71	-	0,51	0,76	-	0,57
Biscoito recheado, doces ou guloseimas	0,71	-	0,50	0,70	-	0,50	0,76		0,57
Feijão		0,36	0,13	-	0,36	0,13	-	0,41	0,17
Frutas frescas		0,58	0,35	-	0,59	0,35	-	0,58	0,35
Verduras e/ou legumes		0,81	0,64	-	0,80	0,64	-	0,82	0,67
Variância ^a^	0,25	0,17	-	0,20	0,17	-	0,28	0,17	-
Índices de ajuste ^b^									
RMSEA (IC 90%)	0,062 (0,061–0,063)			0,062 (0,061–0,064)			0,068 (0,067–0,068)		
TLI	0,94			0,94			0,94		

RMSEA: *root mean square error of approximation* . TLI: *Tucker-Lewis index* . CFI: *comparative fit index* . h ^2^: comunalidade.

^a^ Variância explicada de cada fator (normatizada entre 0 e 1). ^b^ Parâmetros de referência: cargas fatoriais ≥ 0,30 e < 0,85. RMSEA < 0,08. TLI > 0,95.

Os resultados da investigação de invariância de mensuração desta estrutura estão descritos na Tabela 2 , a partir das análises fatoriais confirmatórias multigrupo. Considerando os valores de referência para os índices de ajuste, as condições de invariância configural foram confirmadas, apontando que a estrutura bidimensional, com os respectivos itens em cada fator subjacente ao conjunto de marcadores, guarda equivalência entre as cinco macrorregiões, as diferentes fases do curso da vida e também longitudinalmente, no período de 2015 a 2019.

**Tabela 2:** Evidências de invariância de mensuração para o conjunto de marcadores de consumo alimentar do Sistema de Vigilância Alimentar e Nutricional (Sisvan) entre macrorregiões, fases do curso da vida e ao longo do tempo (2015-2019), Brasil.

Grupos de interesse	Índices de ajuste ^a^					

	RMSEA (IC 90%)	TLI	CFI	Comparação	ΔRMSEA	ΔCFI
Macrorregiões						
A. Invariância configural	0,034 (0,033-0,035)	0,969	0,981			
B. Invariância métrica	0,033 (0,032-0,033)	0,971	0,977	B *vs.* A	-0,001	-0,004
C. Invariância escalar	0,039 (0,039-0,040)	0,959	0,970	C *vs.* B	0,006	-0,007
Fases do curso da vida						
A. Invariância configural	0,027 (0,027-0,028)	0,969	0,985			
B. Invariância métrica	0,026 (0,025-0,027)	0,978	0,983	B *vs.* A	-0,001	-0,002
C. Invariância escalar	0,037 (0,036-0,038)	0,957	0,968	C *vs.* B	-0,011	-0,015
Anos (2015-2019)						
A. Invariância configural	0,032 (0,031-0,032)	0,978	0,987			
B. Invariância métrica	0,028 (0,028-0,028)	0,983	0,986	B *vs.* A	-0,004	-0,001
C. Invariância escalar	0,003 (0,003-0,003)	0,980	0,986	C *vs.* B	0,002	0

RMSEA: *root mean square error of approximation* . TLI: *Tucker-Lewis index* . CFI: *comparative fit index* . ΔRMSEA: diferença entre valores de RMSEA; ΔCFI: diferença entre valores de CFI (na comparação dos modelos B e A e dos modelos C e B).

^a^ Parâmetros de referência: RMSEA < 0,08. TLI e CFI < 0,95. ΔRMSEA < 0,015. ΔCFI < 0,01.

Na sequência, a invariância métrica (modelo B em relação ao modelo A) e a invariância escalar (modelo C em relação ao modelo B) do formulário de marcadores do consumo alimentar foram confirmadas para todos os grupos de interesse ( Tabela 2 ). Esses achados indicaram que os pesos de cada item e sua escala foram homogêneos, apresentando importância equivalente para os fatores relacionados à alimentação saudável e não saudável, independentemente das macrorregiões, das fases do curso da vida e ao longo dos anos.

## DISCUSSÃO

Este estudo reúne evidências originais sobre a estrutura interna do formulário de marcadores do consumo alimentar do Sisvan para indivíduos a partir de 2 anos de idade, provendo, ainda, evidências de invariância de mensuração dos fatores relacionados à alimentação saudável e não saudável do instrumento. Os achados confirmam que os marcadores do consumo alimentar do Sisvan estão bem fundamentados na proposta teórica utilizada para o desenvolvimento dos itens do instrumento e sugerem que sua estrutura interna é estável em diversos recortes investigados.

As recomendações alimentares que guiaram conceitualmente a concepção do formulário de marcadores do consumo alimentar do Sisvan são centradas na problematização do processamento industrial de alimentos segundo a classificação NOVA ^2^ . Sob essa abordagem, alimentos ultraprocessados são indicativos de alimentação não saudável, ao passo que alimentos *in natura* e minimamente processados guardam características pertinentes a práticas alimentares saudáveis ^8^ . Essa perspectiva refletiu-se na estrutura interna do instrumento, pautado no relato do consumo alimentar individual do dia anterior, de forma que quatro itens listando alimentos ultraprocessados compuseram o fator 1 e três itens listando alimentos *in natura* ou minimamente processados compuseram o fator 2, com cargas fatoriais adequadas e ajuste considerado satisfatório. A disposição dos itens segundo essas duas dimensões é pertinente à literatura que demarca a relação de alimentos ultraprocessados com aumento da ingestão energética e consequente ganho de peso ^18^ , bem como as associações com piores desfechos de saúde em diversos grupos populacionais ^19^ , em contraposição aos efeitos associados a dietas ricas em frutas, hortaliças e feijões ^22^ , entre outros alimentos com baixo nível de processamento industrial.

A disseminação da comercialização e disponibilidade domiciliar de alimentos ultraprocessados tem sido registrada em diferentes contextos ao redor do mundo. Análises globais entre 2006 e 2019 sublinharam vendas mais elevadas desses produtos na Europa, Oceania, América do Norte e América Latina, ao passo que rápidos incrementos foram constatados na Ásia, no Oriente Médico e na África ^23^ . No Brasil, esse espalhamento está documentado ao longo das últimas décadas por meio de Pesquisas de Orçamentos Familiares ^24^ . Adicionalmente, sabe-se que existe uma associação inversa entre a aquisição e a disponibilidade domiciliar de alimentos ultraprocessados e aquela de hortaliças, o que se estende ao consumo individual desses itens ^25^ .

Esse panorama pode sustentar as evidências de invariância de mensuração observadas para o formulário de marcadores do consumo alimentar do Sisvan. Com duas dimensões distintas identificadas em sua estrutura interna, houve consistência quanto aos itens atrelados a cada um dos fatores, aos pesos atribuídos aos itens e à igualdade escalar do instrumento entre macrorregiões brasileiras, fases do curso da vida e no período de 2015 a 2019. Sugere-se, assim, um desempenho adequado desse conjunto de marcadores do consumo alimentar sob variados traços culturais relacionados à alimentação; para diferentes faixas etárias e suas correspondentes necessidades nutricionais; e ao longo dos últimos anos no país.

Pode-se dizer que análises aqui apresentadas vêm em uma esteira de discussão quanto ao emprego da classificação NOVA, adotada pelo Sisvan no Brasil no monitoramento de indicadores de alimentação adequada e saudável. Apesar de consistir em uma abordagem factível, levantamentos internacionais sobre fatores associados à morbimortalidade, a exemplo da série de estudos Global Burden of Disease, ainda não incluem alimentos ultraprocessados em seus índices de dieta ^1^ . Em relatório de consulta técnica para mensuração de dietas saudáveis realizada em 2021 ^26^ , a Organização Mundial da Saúde admitiu que pouca atenção tem sido direcionada a abordagens que expandam as avaliações para além de componentes centrados em nutrientes, reconhecendo os desafios mundiais quanto à capacidade para a coleta de métricas de alimentação em larga escala que permitam o monitoramento e instruam adequadamente intervenções populacionais ^26^ .

Os achados deste estudo conferem, assim, envergadura para a qualificação das ações de vigilância alimentar e nutricional no contexto brasileiro, com implicações em ao menos duas camadas. Primeiramente, as evidências podem potencializar o uso dos formulários de marcadores do consumo alimentar do Sisvan em pesquisas epidemiológicas com variados delineamentos e recortes populacionais, como um instrumento adequadamente avaliado quanto às características e à consistência de sua estrutura interna.

É importante enfatizar também a necessidade de ampliação dos esforços de análise dos dados produzidos continuamente nos serviços da atenção primária à saúde do SUS, reconhecendo o Sisvan como um sistema de informação singular e com grande possibilidade de expansão para o acompanhamento da situação alimentar da população brasileira. Sob essa camada, fortalecem-se noções de dupla via para o conceito ampliado de vigilância alimentar e nutricional preconizado na Política Nacional de Alimentação e Nutrição ^6^ .

Em segundo lugar, este estudo contribui para a integração mais informada de ações de vigilância com perspectivas de produção do cuidado e orientação individual de usuários do SUS. Pela primeira vez, dispõe-se de respaldo estatístico para a compreensão da mensuração quantitativa de características da alimentação por meio deste instrumento, o que subsidia mais apropriadamente suas indicações de uso ^10^ . Nesse sentido, destacam-se recomendações recentes do emprego dos marcadores do consumo alimentar do Sisvan em árvores de decisões que estruturam protocolos de uso do Guia Alimentar para a População Brasileira em orientações individuais para a população em geral, em diversas faixas etárias ^27^ , e para pessoas adultas com obesidade, hipertensão arterial e diabetes melito ^28^ .

A interpretação dos resultados apresentados deve ser feita à luz das limitações deste estudo. Por compor uma estratégia de vigilância em saúde já implementada, as presentes análises não se voltaram a etapas de confecção dos itens do instrumento avaliado. Ainda assim, os materiais orientadores do Sisvan ^7^ esclareceram as bases conceituais consideradas à época da proposição do formulário de marcadores do consumo alimentar e assume-se que a listagem dos alimentos elencados tenha compreensão razoavelmente direta. A cobertura populacional do Sisvan pode ser considerada limitada, porém, para as análises de caráter psicométrico conduzidas neste trabalho, ressalta-se que as amostras analíticas supriram adequadamente os requisitos mínimos em todos os recortes explorados.

Como pontos fortes, devem-se notar a amplitude nacional das análises e a disponibilidade de dados de usuários dos serviços de saúde, que responderam ao instrumento nas situações de uso para as quais este foi concebido. Espera-se que essas evidências potencializem esforços para a ampliação da cobertura da avaliação de marcadores do consumo alimentar no SUS. A fim de aprofundar a compreensão deste formulário, perspectivas adicionais de validação podem incluir a investigação do perfil nutricional de alimentos consumidos, bem como disponíveis em âmbito domiciliar, de forma atrelada à apuração do conjunto de marcadores do consumo alimentar.

## CONCLUSÃO

A estrutura interna do formulário de marcadores do consumo alimentar do Sisvan para indivíduos a partir de 2 anos de idade refletiu seu embasamento conceitual a partir da classificação NOVA e das recomendações do Guia Alimentar para a População Brasileira. Dois fatores distintos agruparam alimentos ultraprocessados e alimentos *in natura* ou minimamente processados, de forma que o instrumento se mostrou estável em configuração, cargas fatoriais e escala entre as macrorregiões brasileiras, fases do curso da vida e ao longo do tempo. A estrutura interna e as evidências de invariância de mensuração constatadas subsidiam o uso amplo dos marcadores do Sisvan para monitoramento do consumo alimentar.
